# Desert Physio-Ecological Adaptation of *Amorpha fruticosa* to Dynamic Shading Under Photovoltaic Panels in a Sandy Region

**DOI:** 10.3390/plants15050717

**Published:** 2026-02-27

**Authors:** Lu Liu, Ruidong Wang, Yong Gao, Yifang Su

**Affiliations:** 1 State Key Laboratory of Water Engineering Ecology and Environment in Arid Area, Inner Mongolia Agricultural University, Hohhot 010000, China; 15034818270@163.com (L.L.); 18830666583@163.com (Y.S.); 2 College of Desert Control and Engineering, Inner Mongolia Agricultural University, Hohhot 010000, China

**Keywords:** photovoltaic power station, *Amorpha fruticosa*, shading, photosynthetic physiology, growth characteristics

## Abstract

The construction of photovoltaic (PV) power stations for sand control in northwestern China has exacerbated the conflict between solar resource utilization and ecosystem fragility, creating urgent ecological challenges that demand immediate solutions. This study investigated *Amorpha fruticosa* growing under fixed adjustable PV panels at the CGN DaLate Photovoltaic Leading Base in the eastern hinterland of the Kubuqi Desert. Through long-term field observations, three shading time gradients were established: heavy shading (HS), light shading (LS), and no shading (CK, control). The results clearly demonstrated that: (1) Plants in the LS treatment exhibited significantly greater plant height, basal diameter, and crown width compared to those in HS and CK, indicating optimal growth status and morphological plasticity. They maintained the highest net photosynthetic rate (Pn) and water use efficiency (WUE), while their intercellular CO_2_ concentration (Ci) was significantly lower than in CK, effectively mitigating photosynthetic inhibition caused by high light intensity. Total chlorophyll (Chl) content increased significantly with increasing shading intensity, whereas the Chl a/b ratio decreased. (2) The LS treatment yielded the highest nitrogen (N), phosphorus (P), and crude protein (CP) contents, along with a more balanced N:P ratio, suggesting a superior state of nutritional metabolism. Growth indicators showed significant positive correlations with WUE and Chl content, and significant negative correlations with transpiration rate (Tr) and Ci, confirming a synergistic “physiological adaptation-growth optimization” mechanism. Our results demonstrate that light shading represents the optimal condition for the growth and biomass accumulation of *A. fruticosa*, highlighting its potential as a key species for vegetation restoration in PV power stations within arid ecosystems. These findings not only elucidate the plant’s adaptation mechanisms but also provide a crucial physiological basis for selecting and managing understory vegetation, thereby supporting the optimization of integrative “PV-Ecology” systems for sustainable desert restoration.

## 1. Introduction

Against the backdrop of increasingly severe global climate change and the ongoing promotion of the “Dual Carbon” strategy, transitioning the energy structure towards clean and low-carbon sources has become a universal consensus within the international community [1]. In this process, photovoltaic (PV) power generation, as a mature renewable energy technology, has achieved large-scale application worldwide due to its advantages of abundant resources, wide distribution, and environmental friendliness [2]. China, a global leader in the PV industry, has seen its installed capacity grow continuously, exceeding 1.1 billion kilowatts by July 2025 [3]. PV power generation is a vital pathway for promoting clean energy transition and ensuring national energy security. The integrated model of “power generation above panels and ecological restoration below panels” fosters synergistic benefits, promoting green economic growth and enabling deep integration between energy infrastructure and ecological environment management [4,5]. Despite the theoretical promise of this synergistic model, its practical application in fragile desert ecosystems faces significant ecological obstacles that must be addressed to realize these benefits.

The vast desert and sandy lands of northwestern China present ideal conditions for large-scale PV installations, characterized by arid climates, prolonged sunshine, high solar radiation, and extensive tracts of low-cost, flat terrain. However, the ecological impacts of such development require urgent attention. The construction and operation of large-scale PV arrays may exacerbate soil erosion, interfere with vegetation recovery, and impose additional pressure on the original desert ecosystem [6,7,8]. The moving shadows cast by PV modules create high spatiotemporal heterogeneity in environmental factors such as light, temperature, and water. This dynamic shading effect, coupled with the physical interception of precipitation by PV panels that reduces soil water replenishment beneath the arrays, may lead to ecological issues like surface drying and vegetation degradation, thereby constraining the sustainable development of the PV industry [9].

In the new era of digital energy, the green and low-carbon transition is progressing steadily. The “PV + sand control” model, as an innovative approach to desertification management [10], is developing rapidly. Nevertheless, the ecological issues induced by environmental changes remain a critical challenge, continuously intensifying the contradictory effects between the geographical advantage of PV resources in sandy areas and the fragility of the ecological environment [11]. Selecting suitable understory plant species with both shade tolerance and sand-fixing capacity is key to the success of the “PV + sand control” ecological model [12,13]. *A. fruticosa*, a pioneer species with strong stress resistance, can effectively reduce wind speed, impede sand movement, and improve sandy soil through biological nitrogen fixation, demonstrating significant potential for sand control [14,15]. Existing research has primarily focused on the drought resistance of vegetation types in sandy areas. Systematic studies on the photosynthetic physiological responses and adaptation mechanisms of plants under the dynamic shading habitats created by PV panels are still lacking, which to some extent limits the scientific application and benefit evaluation of this species in “PV sand control” projects.

This study was conducted at the CGN DaLate PV Leading Base in the eastern hinterland of the Kubuqi Desert, focusing on *A. fruticosa* growing beneath fixed PV panel arrays. We hypothesized that within the dynamic shading habitat created by PV arrays, an intermediate shading intensity (light shading) would optimize the photosynthetic performance and water-use balance of *A. fruticosa*, thereby maximizing its growth and nutrient accumulation, rather than heavier shading or full sunlight conditions. To test this hypothesis, three shading time gradients were established based on field observations: heavy shading (HS), light shading (LS), and no shading (CK, control). We systematically examined the responses of *A. fruticosa* in terms of growth, photosynthetic parameters, chlorophyll content, and nutrient composition to these dynamic shading conditions. The research aimed to elucidate the plant’s adaptation strategies to intermittent PV shading, thereby providing a scientific basis and practical guidance for selecting understory vegetation and supporting ecological restoration efforts in sandy-area PV power stations.

## 2. Results

### 2.1. Growth Characteristics of A. fruticosa Under PV Shading Gradients

Different shading gradients significantly affected all growth indicators of *A. fruticosa* (Table 1). *A. fruticosa* exhibited enhanced growth under shading treatments compared to the full-sun control (CK) throughout the growing season, with the Light Shading (LS) treatment consistently yielding the highest values for plant height, basal diameter, and crown width (Table 1). In September, for instance, plant height under LS (59.33 cm) surpassed that of HS and CK by 28.0% and 43.5%, respectively. Basal diameter (1.25 cm) and crown width (66.17 cm) in LS were also the largest, indicating that the light shading environment was most favorable for biomass accumulation in *A. fruticosa*.

The response of leaf morphological indicators to shading was more complex. At the beginning of the growing season, leaf length and width in both shading zones (HS, LS) were significantly higher than in CK, suggesting that shading prompted plants to increase leaf area to enhance light capture. As the plants grew, leaf dimensions generally decreased across all shading zones. By September, only leaf length in LS (2.36 cm) remained significantly higher than in CK (1.79 cm), while leaf width showed no significant differences among zones. This change might be related to leaf senescence and resource reallocation induced by intensified drought stress later in the season. Leaf thickness followed a different pattern. Throughout the observation period, leaves in CK were thicker. While leaf thickness in HS and LS showed no significant difference in July, a trend emerged from August onwards where leaves in HS (0.14 mm) were thinner than those in LS (0.16 mm). By September, a stable gradient of “CK > LS > HS” was established. This indicates that under the dual stress of full sun and drought in CK, *A. fruticosa* tended to develop thicker leaves, a typical xeromorphic adaptation. Under shading conditions, leaves became thinner, exhibiting characteristics of shade-adapted plants.

### 2.2. Effects of PV Shading on Photosynthetic Characteristics of A. fruticose

**Figure 1:**
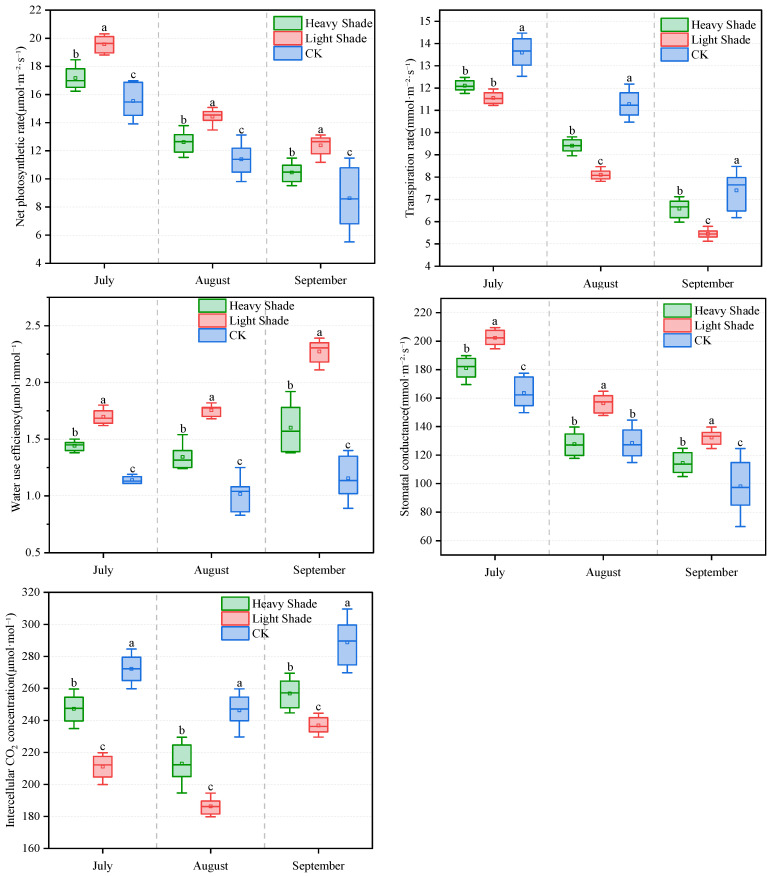
Photosynthetic parameters of *A. fruticosa* under different shading gradients. Different lowercase letters indicate significant differences among treatments at the same time point (*p* < 0.05).

Photosynthetic physiological responses of *A. fruticosa* differed significantly among shading gradients (Figure 1). Throughout the observation period, leaves in the LS treatment consistently maintained a relatively high net photosynthetic rate (Pn). In July, the mean Pn in LS (19.58 μmol·m^−2^·s^−1^) was significantly higher than in HS (17.51 μmol·m^−2^·s^−1^) and CK (15.54 μmol·m^−2^·s^−1^) (*p* < 0.05). Although Pn decreased across all shading zones as the growing season progressed, LS maintained a clear advantage in August and September (14.32 and 12.38 μmol·m^−2^·s^−1^, respectively). The light shading environment effectively mitigated photosynthetic inhibition caused by summer high light and drought stress. Consistent with Pn trends, water use efficiency (WUE) in LS was significantly the highest across all three months, reaching 2.27 μmol·mmol^−1^ in September, approximately twice that of CK (1.15 μmol·mmol^−1^). This indicates a physiological advantage in water use efficiency under arid conditions. Transpiration rate (Tr) in CK was the highest throughout the observation period. Combined with its lower Pn, this resulted in the lowest WUE. LS, while maintaining relatively high photosynthetic capacity, exhibited significantly lower Tr, achieving a better balance between water consumption and carbon assimilation. Stomatal conductance (Gs) changes largely mirrored Pn trends, with LS maintaining the highest values each month, while CK showed lower and more variable Gs (e.g., standard error of 19.8 in September), reflecting stomatal instability under full sunlight. Intercellular CO_2_ concentration (Ci) showed a trend opposite to Pn: CK > HS > LS. In September, the mean Ci in CK was as high as 288.8 μmol·mol^−1^, significantly higher than in LS (236.8 μmol·mol^−1^). The higher Ci in CK suggests a lower rate of CO_2_ consumption within the leaf, which, combined with other parameters, indicates that non-stomatal limitations might have been stronger in CK by the end of the growing season [16].

In summary, LS created a “light-water coordinated” micro-environment for *A. fruticosa* in the Kubuqi Desert. This environment not only ensured sufficient photosynthetic drive but also significantly improved water use efficiency by reducing transpirational water loss, leading to optimal performance in both net carbon production and drought resistance. Heavy shading (HS) limited photosynthetic potential due to insufficient light energy, while CK suffered from the dual stress of high light and drought, resulting in the lowest and most variable photosynthetic efficiency. 

### 2.3. Effects of PV Panel Shading on Chlorophyll Content in A. fruticosa Leaves

**Figure 2:**
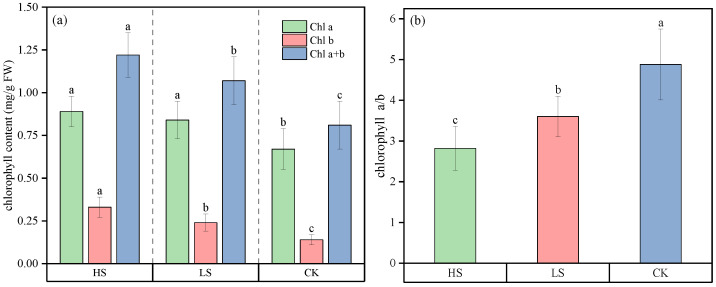
Chlorophyll content of *A. fruticosa* leaves throughout the growing season (July to September) under different shading gradients (mg/g). (**a**) Chlorophyll a (Chl a), chlorophyll b (Chl b), and total chlorophyll (Chl a+b) content; (**b**) Chlorophyll a/b ratio. Data are presented as Mean ± Standard Deviation of measurements from the entire growing season (*n* = 5 repetitions × 3 months). Different lowercase letters indicate significant differences in the same measurement index under different shading gradients (*p* < 0.05).

Throughout the growing season (July–September), significant differences were observed in the chlorophyll content and its components in *A. fruticosa* leaves under different shading gradients (Figure 2). In terms of total chlorophyll (Chl) content, HS exhibited the highest value (1.22 mg/g), followed by LS (1.07 mg/g), and CK the lowest (0.81 mg/g), showing a consistent increasing trend with prolonged shading duration. This indicates that shading significantly promotes chlorophyll biosynthesis. The trends of Chl a and Chl b contents were generally consistent with that of total Chl, both reaching their highest levels under HS and the lowest under CK. Notably, Chl b was more sensitive to shading: the Chl b content in HS (0.33 mg/g) was approximately 2.36 times that in CK (0.14 mg/g), whereas the increase in Chl a was relatively smaller. This compositional difference was directly reflected in the Chl a/b ratio, which was highest in CK (4.88), followed by LS (3.60), and lowest in HS (2.82), exhibiting a systematic decline with increasing shading intensity. This suggests that under low-light conditions, plants optimize the light capture efficiency of Photosystem II by relatively increasing the proportion of Chl b, thereby adapting to changes in the light environment [17]. Overall, shading not only increased total chlorophyll content but also adjusted the compositional ratio of photosynthetic pigments, representing an important light adaptation strategy for plants under low-light stress.

### 2.4. Effects of PV Panel Shading on Nutrient Composition of A. fruticosa

**Figure 3:**
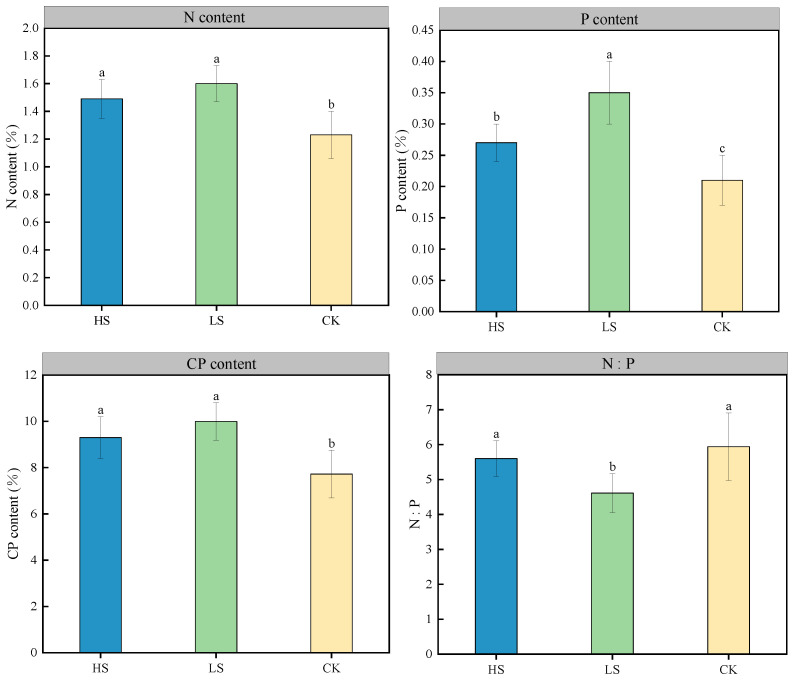
Leaf nutrient content of *A. fruticosa* under different shading gradients throughout the growing season (July to September) (%). Data are presented as Mean ± Standard Deviation of measurements from the entire growing season (*n* = 5 repetitions × 3 months). Different lowercase letters indicate significant differences in the same measurement index under different shading gradients (*p* < 0.05).

The results of nitrogen (N), phosphorus (P), and crude protein (CP) contents in *A. fruticosa* leaves under different shading gradients (Figure 3) indicate that PV panel shading significantly affects plant N and P nutrient metabolism. Based on the mean values across the entire growing season, LS exhibited the optimal levels in N, P, and CP contents, with N content (1.60%), P content (0.35%), and CP content (9.99%) being significantly higher than those in CK (1.23%, 0.21%, and 7.72%, respectively), while HS (1.49%, 0.27%, and 9.30%) fell between the two treatments. This result suggests that moderate shading facilitates the synthesis and accumulation of nitrogen and protein in plants, while also promoting phosphorus uptake and utilization to a certain extent. Further analysis of the N:P ratio revealed that LS maintained the most balanced ratio, with a growing-season mean of 4.61, significantly lower than those of CK (5.94) and HS (5.60). The higher N:P ratio in CK indicates a tendency toward relative nitrogen accumulation under full-light, non-shaded conditions, whereas the more balanced N:P ratio in LS is closer to the optimal stoichiometric range for plant growth. PV panel shading not only affects the absolute contents of N and P nutrients but also regulates the balance between them. Among the treatments, light shading (LS) is most conducive to maintaining high protein synthesis potential and a coordinated N:P nutrient structure.

### 2.5. Comprehensive Analysis of Growth and Physiological Status of A. fruticosa in Different PV Inter-Row Shading Zones

**Figure 4:**
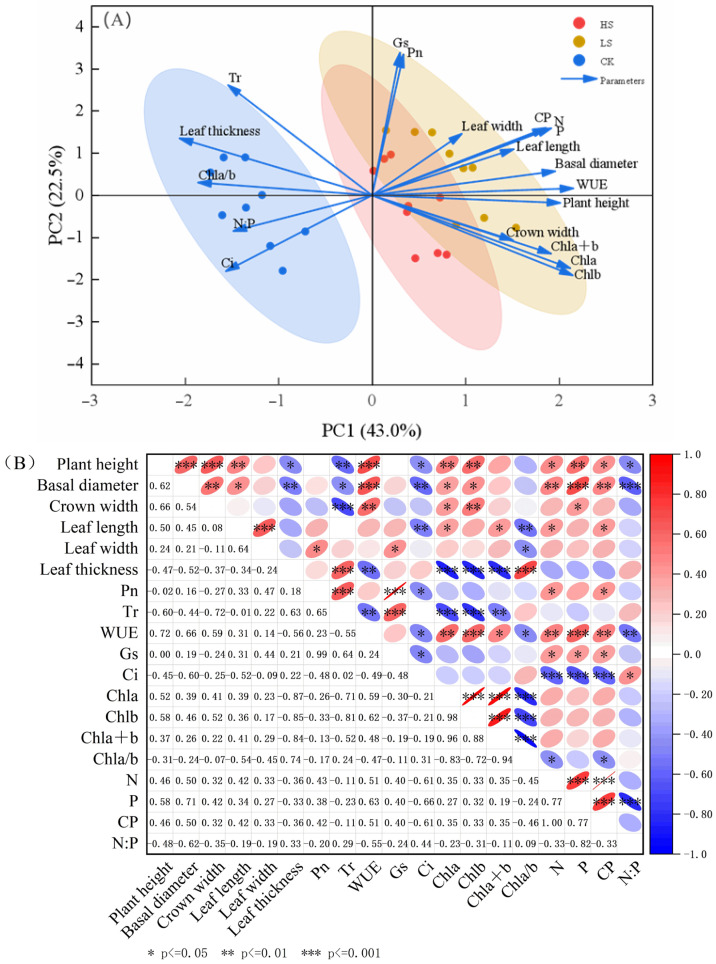
Comprehensive analysis of growth and physiological indicators of *A. fruticosa.* (**A**) Principal component analysis, (**B**) Correlation analysis.

Principal Component Analysis (PCA) results (Figure 4A) showed that the cumulative contribution rate of the first two principal components (PC1 and PC2) reached 65.5%, effectively capturing the variation characteristics of *A. fruticosa* growth and physiological indicators. Based on the loading plot, PC1 exhibited strong positive loadings for key growth and photosynthetic indicators, including net photosynthetic rate (Pn), stomatal conductance (Gs), water use efficiency (WUE), plant height, basal diameter, and crown width, while showing negative loadings for intercellular CO_2_ concentration (Ci) and the chlorophyll a/b ratio (Chl a/b). This suggests that PC1 primarily integrates information related to “photosynthetic assimilation–growth accumulation” and represents a comprehensive indicator of the growth performance and photosynthetic efficiency of *A. fruticosa*. PC2 was more closely associated with leaf morphological traits (leaf length, width, and thickness) and chlorophyll components, reflecting variation related to the plant’s morphological and physiological adaptation to the light environment.

The distribution of samples from different shading treatments in the principal component space showed distinct clustering patterns. Samples from the light shading (LS) treatment clustered in the high-positive region of PC1, whereas those from the heavy shading (HS) treatment were located in the positive region of PC2. Control (CK) samples were mostly distributed in the negative region of PC1 and the low-to-mid range of PC2. This spatial pattern visually indicates that light shading was most conducive to the synergistic enhancement of growth and photosynthesis in *A. fruticosa*. Correlation analysis further revealed the internal relationships among indicators (Figure 4B). Plant height, basal diameter, and crown width showed significant positive correlations with WUE, Chl a, and Chl b (*p* < 0.05), and significant negative correlations with Tr (*p* < 0.05). This indicates the material foundation provided by Chl accumulation for early growth and the formation of a “low consumption-high utilization” water adaptation pattern. Leaf length showed significant positive correlations with Chl a, Chl b, and total Chl content. Leaf thickness showed significant negative correlations with WUE, Chl a, Chl b, and total Chl content, and a significant positive correlation with Tr. This reflects a xeromorphic adaptation strategy to cope with high light and drought stress. N, P, and CP contents showed significant positive correlations with plant height and crown width; the N:P ratio showed significant negative correlations with plant height and basal diameter, reflecting the regulatory role of nutrient element balance on plant growth.

## 3. Discussion

### 3.1. Regulation of Growth Morphology and Habitat Adaptability of A. fruticosa by PV Panel Shading

Our results demonstrate that PV panel shading significantly reshapes the growth morphology of *A. fruticosa*, with the degree of shading determining the outcome. Plasticity in plant growth morphology is a crucial strategy for adapting to variable environments. PV panel shading directly influences the growth patterns of *A. fruticosa* by altering micro-environmental factors such as light, temperature, and water [18]. This study found that plant height, basal diameter, and crown width of *A. fruticosa* under LS were significantly superior to those under HS and CK, with September plant height being 43.5% higher than CK. This aligns with findings by Marrou H et al. [19] in agrivoltaic systems, where “moderate shading alleviates environmental stress and promotes plant growth.” The underlying mechanism for this phenomenon lies in the fact that the moderate shading created by PV arrays establishes a critical “stress buffer zone” in the arid Kubuqi Desert. The Kubuqi Desert is arid with low precipitation and high evaporation. The entire growing season exhibits distinct seasonal variations: July is characterized by high temperatures and limited rainfall, exposing plants to the most intense light and heat stress; August maintains high temperatures but may be accompanied by minor precipitation, marking the onset of a vigorous growth period; September sees a decline in temperature, but drought intensifies. *A. fruticosa* in CK faced dual stress from high light and drought, leading to thickened leaves (a xeromorphic adaptation) but restricted growth. This is consistent with observations by Zhao Yan et al. [20] regarding growth inhibition in *A. fruticosa* under water stress. Conversely, HS, due to insufficient light resources, exhibited thinner leaves as an adaptation to low light, but insufficient photosynthetic product accumulation resulted in weaker growth performance compared to LS. The LS environment strikes a balance between these two extremes. It reduces the intense solar radiation responsible for photoinhibition and high transpirational water loss, yet maintains adequate photosynthetically active radiation for photosynthesis. This balanced, “light-water coordinated” microenvironment favors a shift in resource allocation within *A. fruticosa*, prioritizing “growth investment” over “stress defense,” which is ultimately reflected in its superior morphological development.

The superior performance of *A. fruticosa* under LS can also be interpreted from an evolutionary and ecological perspective. This species naturally occurs in riparian zones and forest edges—habitats characterized by dynamic light regimes, intermittent shading from canopy gaps, and fluctuating solar angles [21]. Over evolutionary time, *A. fruticosa* has acquired adaptive traits such as high morphological plasticity, efficient light-harvesting capacity, and optimized water-use strategies to exploit such heterogeneous light environments. Notably, the PV shading environment, particularly under LS, effectively simulates this native light niche by creating a shifting mosaic of sun and shade patches driven by the diurnal movement of the panels and the sun. This ecological resemblance likely triggers pre-adapted physiological and morphological responses in *A. fruticosa*, enabling it to perform optimally under LS [22]. Hence, the success of *A. fruticosa* in desert PV stations is not merely a case of stress alleviation, but also a reflection of its evolutionary heritage—a strong ecological rationale for its selection as a keystone restoration species in these engineered habitats.

Differentiated leaf morphology further reflects the habitat adaptation strategies of *A. fruticosa*. In the early growing season, leaf length and width in shaded zones were significantly larger than in CK, a typical adaptation where plants increase light-receiving area to enhance capture efficiency under low light [23]. In September, the size of leaves from all treatments generally decreased, which was mainly related to the intensified drought stress at the end of the growing season and the transfer of nutrients to the stems. This link between “morphological plasticity-environmental adaptation” has similarly been observed in Phoebe zhennan seedlings studied by Dai Dachuan et al. [24], suggesting the regulation of leaf morphology in arid zone shrubs by shading has generality. Notably, leaf thickness in LS was intermediate between CK and HS. This avoided the limitation on photosynthetic gas exchange imposed by the xeromorphic structure in CK, while also preventing excessive water loss due to overly thin leaves as in HS. This balanced morphological state forms an important structural basis for the optimal growth of *A. fruticosa* under the dynamic light environment of PV arrays, which directly informs optimal planting positions within the inter-row space [25].

### 3.2. Coordinated Effects and Limiting Factors of Photosynthesis and Nutrient Metabolism in A. fruticosa

The ability of *A. fruticosa* to adapt to the PV shading environment relies on active adjustments in its photosynthetic physiological traits. The results clearly show that LS consistently maintained the highest Pn and WUE, with September WUE being approximately twice that of CK. This advantage is particularly prominent at the end of the growing season: September marks a period when temperatures in the Kubuqi Desert decline but drought intensifies, making water the primary limiting factor for photosynthesis. Through efficient water use strategies, LS plants maintain a high carbon assimilation capacity under limited water conditions, thus exhibiting significant physiological advantages during seasonal drought. The key mechanism lies in the effective regulation of the canopy microclimate by PV shading: on the one hand, it lowers leaf temperature and air vapor pressure deficit, thereby reducing stomatal transpiration; on the other hand, the moderate diffuse light maintains the efficient operation of Photosystem II (PSII) [26]. This contrasts with the photosynthetic inhibition mechanism observed by Liu J et al. [27] under drought stress: in CK, sustained high light caused damage to the photosynthetic apparatus (manifested as increased Ci), representing a non-stomatal limitation. In contrast, LS achieved an efficient balance between carbon assimilation and water consumption through optimized stomatal behavior and protection of the photosynthetic machinery. This regulatory strategy is crucial for plants adapting to resource-scarce environments in arid zones, echoing the photosynthetic-water coordination mechanism observed in *Haloxylon ammodendron* in deserts [28].

Changes in Chl content and composition further reveal the low-light adaptation mechanism. From the perspective of the mean values throughout the growing season, the increase in shading intensity led to an elevation in total chlorophyll content and a decrease in the chlorophyll a/b ratio, with chlorophyll b being more sensitive to shading conditions: the mean chlorophyll b content in HS was approximately 2.35 times that in CK. This aligns with findings by Wan Y et al. [29] in *Paeonia* species, which demonstrated that a relative increase in the Chl b proportion enhances the capture of diffuse light and energy transfer efficiency under low light conditions. This adaptation is rooted in the primary association of Chl b with Light-Harvesting Complex II (LHCII), representing a conserved physiological strategy for optimizing light use under limited resources. However, HS, despite having the highest Chl content, did not exhibit the best photosynthetic performance. This is likely because light intensity fell below the light compensation point, leading to insufficient photosynthetic product accumulation, consistent with the “shading threshold effect” proposed by Bayrak F et al. [30]. When shading duration exceeds a critical threshold, light limitation outweighs the advantage of pigment accumulation. In this study, the monthly average shading duration for HS was 9 h 18 min, which likely exceeded the suitable light intensity range for *A. fruticosa*. This critical value is similar to the finding by Touil S et al. [31] for horticultural crops, where “6–8 h of shading is the suitable range,” further confirming that the regulation of plant photosynthetic performance by shading gradients exhibits a threshold effect.

The differential response in nutrient metabolism provided material support for the growth of *A. fruticosa*. From the perspective of the mean values throughout the growing season, LS had the highest N, P, and CP contents, and the average nitrogen to phosphorus ratio (N:P) reached a suitable proportion of 4.61. This aligns with the nutrient accumulation pattern observed in alfalfa under agrivoltaic systems by Edouard S et al. [32]. This balanced nutritional status synergistically interacts with efficient photosynthesis: ample photosynthetic products provide the carbon skeleton for nitrogen assimilation and protein synthesis, while the optimized N:P ratio ensures the efficient functioning of the photosynthetic enzyme system (e.g., *Rubisco*). As a nitrogen-fixing plant, shading may improve soil moisture conditions, promoting rhizobial activity and thereby enhancing nitrogen uptake efficiency in *A. fruticose* [14]. The increase in P content might be related to reduced soil evaporation and phosphorus leaching under shading [33]. It is noteworthy that CK exhibited a significantly higher N:P ratio (mean value 5.94) throughout the entire growing season. Such nutritional imbalance might limit the synthesis of photosynthesis-related enzymes, further exacerbating photosynthetic inhibition, consistent with the view proposed by Sun Q et al. [34] that “nutrient balance is a key regulatory factor for plant growth.”

### 3.3. Application Value and Optimization Directions for A. fruticosa in Desert PV Ecological Restoration

This study confirms that *A. fruticosa* possesses comprehensive potential to adapt to the dynamic shaded habitat of desert PV power stations. Its synergistic effect of “morphology-photosynthesis-nutrition” makes it an excellent candidate plant for the PV sand control model. Compared to herbaceous plants, *A. fruticosa* offers advantages such as a robust root network, nitrogen-fixing capacity, and significant windbreak and sand-fixing effects [14], rendering it irreplaceable in the “under-panel restoration, inter-panel planting” strategy. This aligns with the conclusion by Qu Zhun et al. [35] that “perennial shrubs are core vegetation for ecological restoration in desert PV areas.” Our results identify the light shading (LS) condition as optimal for the growth of *A. fruticosa*. This corresponds to a quantifiable shading duration of approximately 6 h per day, which provides a critical design criterion for spatially configuring PV arrays and their under-panel vegetation to concurrently maximize plant productivity and PV energy yield. Therefore, adjusting panel spacing and tilt angle to approximate this optimal shading regime presents a practical strategy for achieving synergy between power generation and ecological restoration in desert PV power stations.

Existing research has primarily focused on the drought resistance of *A. fruticosa*. This study supplements its shading adaptation mechanisms, enriching the physio-ecological research system for suitable plants in arid zones. Compared to plants like *Rehmannia glutinosa* [9] and Phoebe zhennan [24], *A. fruticosa* exhibits more prominent comprehensive adaptive capacity under the dual stress of shading and drought. This is closely related to its characteristics, including nitrogen fixation for soil improvement, strong leaf morphological plasticity, and high water use efficiency. Combined with research by Meng R et al. [18] on the positive impacts of typical desert PV scenarios in China on the growth of sand-adapted plants, the promotion and application of *A. fruticosa* could further enhance the ecological benefits of the “PV sand control” model.

To maximize the role of *A. fruticosa* in PV ecological restoration and achieve synergistic benefits between energy production and ecological protection, the following optimization strategies are proposed based on the shading adaptation mechanisms identified in this study and the habitat characteristics of desert PV areas: (1) Optimization of PV Configuration and Vegetation Spatial Layout: Based on the finding of an optimal shading duration (approximately 6 h/d) for *A. fruticosa*, PV array parameters should be precisely regulated: Employ south-facing fixed-tilt PV array layouts, calculating the minimum inter-row spacing based on solar altitude angles to avoid inter-array shading affecting power generation efficiency while leaving sufficient space for plant growth. Shrubs tolerant of high light and low water consumption, such as *Artemisia ordosica*, can be considered for the fully exposed areas (CK zone), while *A. fruticosa* is recommended for large-scale planting in the inter-row spaces (LS/HS zones). Shade-tolerant herbaceous species can be planted under the panels, constructing a “shrub-grass” composite community. (2) Optimization of Coordinated Nutrient and Water Resource Management: Focus on applying efficient phosphorus fertilizers to stabilize the N:P ratio between 4.0 and 5.0, while reducing the application rates of nitrogen and conventional phosphorus fertilizers. Promote water-saving drip irrigation, utilizing treated cleaning wastewater for irrigation, and dynamically adjusting irrigation frequency and volume based on precipitation patterns. (3) Optimization of Dynamic Monitoring and Regulation: Deploy sensors for light, soil temperature, and moisture. Regularly assess growth and photosynthetic indicators of *A. fruticosa*. Make fine adjustments to panel parameters, water-fertilizer plans, and planting strategies as needed to ensure the synergy between vegetation ecological functions and power station operation.

In summary, *A. fruticosa* demonstrates good potential for adapting to the dynamic shaded habitats in desert PV areas. Under light shading conditions, its growth performance, photosynthetic efficiency, and nutritional status were optimal. The results of this study clarify the application value of *A. fruticosa* in the “PV + sand control” model, providing key physiological parameters and a scientific basis for vegetation configuration and habitat management in desert PV power stations, specifically for “under-panel restoration and inter-panel planting.” This holds significant practical importance for promoting the sustainable development of “PV-ecology synergy.” Future research could expand to screening different functional plant types and optimizing the ecological aspects of PV system configurations to comprehensively enhance ecosystem service functions and overall benefits in power station areas.

## 4. Materials and Methods

### 4.1. Study Area Overview

**Figure 5:**
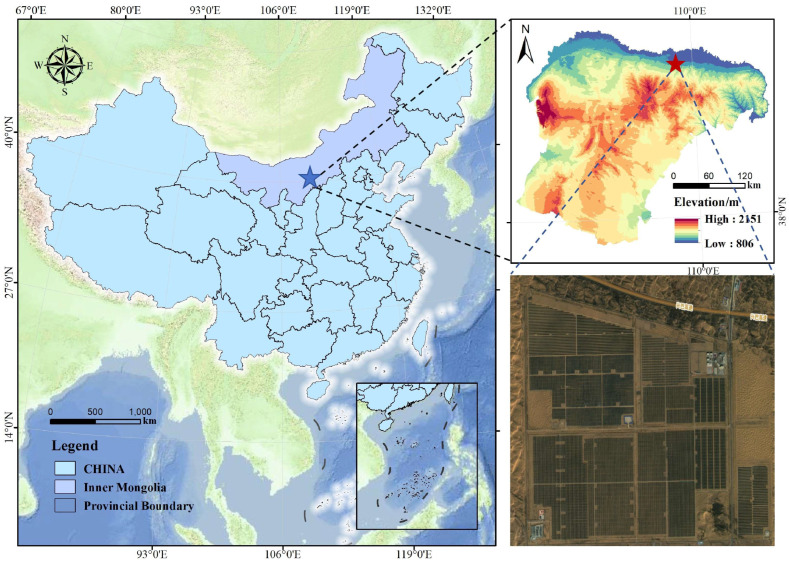
Overview map of the research area.

This study was conducted at the 100 MW No. 3 PV Power Station of the CGN Dalate Photovoltaic Leading Base, located in the eastern hinterland of the Kubuqi Desert within Dalat Banner, Ordos City, Inner Mongolia Autonomous Region, China (Figure 5). The site coordinates are approximately 40.51° N, 109.93° E. The area has a temperate continental climate, characterized by low and variable precipitation. The mean annual precipitation is about 225.6 mm, while potential evaporation ranges from 2100 to 2700 mm. The soil is predominantly sandy, with low nutrient content and poor structural stability. The main vegetation type consists of psammophytic plants, with drought-tolerant and sand-adapted shrubs and herbs such as *Artemisia ordosica* Krasch., *Artemisia sieversiana* Ehrhart ex Willd., *Hedysarum mongolicum* Turez, *Caragana korshinskii* Kom, and *Caragana tibetica* Kom as the dominant species.

The power station commenced operation in 2018 and has been running for seven years, with the regional ecosystem having reached a relatively stable state. Fixed adjustable PV arrays oriented east–west with a south-facing azimuth were selected for observation. A single array consists of 13 panels connected in series. Each panel measures 1300 cm (length) × 420 cm (width), with a tilt angle of 15°. The vertical distance from the rear panel edge to the ground is 270 cm, and from the front edge is 205 cm. The distance between adjacent panels in the north–south direction is 570 cm.

For ecological restoration purposes, the power station established dense planting belts of *A. fruticosa* in May 2021. This species was selected not only for its proven capacity in biological nitrogen fixation and wind-speed reduction, but also for its broad ecological amplitude and extensive global distribution, ranging from North America to multiple regions across Eurasia. As a well-established resilient pioneer species widely employed in international land reclamation programs, *A. fruticosa* exhibits strong adaptability to the harsh environmental conditions of the Kubuqi Desert, offering distinct advantages over many native psammophytes in terms of stress tolerance and rapid establishment. Six rows were planted within the inter-row space between every two PV arrays, running east–west parallel to the arrays. An equidistant planting pattern was adopted, with a row spacing of approximately 65 cm and a plant spacing of about 50 cm, resulting in an estimated planting density of 30,000 plants per hectare. The vegetation relied primarily on natural precipitation, supplemented by equal amounts of drip irrigation during key growth stages each year to ensure consistent water supply across all treatment areas.

### 4.2. Experimental Design

#### 4.2.1. Sample Selection Criteria

**Figure 6:**
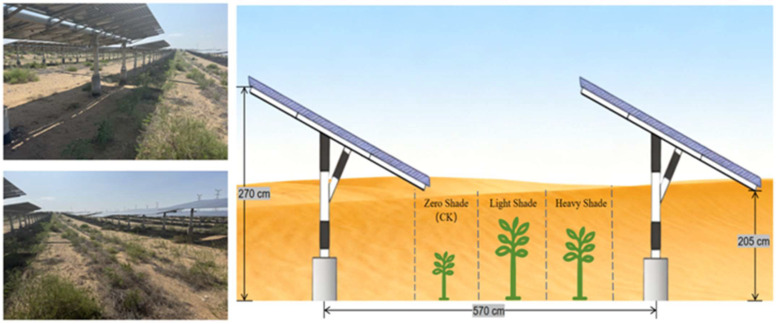
Schematic diagram of *A. fruticosa* planting.

To investigate the growth and physiological characteristics of *A. fruticosa*, three parallel PV arrays in the central area were selected as experimental zones during the 2025 growing season (July–September). Within the inter-row space of each array, three zones were delineated based on light exposure differences (Figure 6): the Heavy Shading (HS) zone, where plants near the panel rear receive limited direct sunlight; the Light Shading (LS) zone, located slightly farther from the panel rear and subject to intermittent shading; and the No Shading (CK) zone, situated in the open area in front of the panels, receiving full sunlight without obstruction throughout the day. Five typical plants with consistent growth vigor were selected from each shading zone within each of the three parallel arrays, resulting in a total of 45 samples (3 arrays × 3 zones × 5 plants). Plant height, leaf length, leaf width, and other growth metrics were measured monthly. Leaf samples were collected between the 12th and 15th of each month, sealed, transported to the laboratory under cool conditions, and stored refrigerated. After removing the midrib, fresh leaf samples were weighed according to the requirements for each physiological measurement. Laboratory analyses were completed within 7 days after sampling.

#### 4.2.2. Shading Duration Calculation

Field observation was employed to calculate shading duration. Prior to the main experiment, two clear, cloudless days were selected. Observations were conducted from 07:00 to 19:00 to record shading conditions in the inter-row zones. The observed average daily shading durations for July were 8 h 25 min for HS and 5 h 10 min for LS; for August, 9 h 20 min for HS and 6 h 25 min for LS; and for September, 10 h 10 min for HS and 6 h 50 min for LS. The monthly average shading durations were calculated as 9 h 18 min for HS, 6 h 8 min for LS, and 0 h for CK, establishing a gradient of HS > LS > CK.

### 4.3. Growth Indicator Measurements

Growth indicators of *A. fruticosa* were measured as follows: Plant height was measured as the vertical distance from the ground to the highest point of the plant using a measuring tape (accuracy 0.1 cm). Basal diameter was measured at 5 cm above ground on the main stem using a digital vernier caliper (accuracy 0.01 mm). Crown width was measured by recording the maximum width of the canopy in the east–west and north–south directions with a measuring tape, and the average was calculated (accuracy 0.1 cm). Leaf length, leaf width, and leaf thickness were measured on healthy, fully expanded, undamaged leaves from the 3rd to 5th node in the middle-upper part of the plant using a digital vernier caliper (accuracy 0.01 mm). Three replicates were taken per leaf, and the mean was calculated.

### 4.4. Physiological Indicator Measurements

#### 4.4.1. Photosynthetic Gas Exchange Parameters

On clear days between 09:00 and 11:00, net photosynthetic rate (Pn), stomatal conductance (Gs), intercellular CO_2_ concentration (Ci), and transpiration rate (Tr) were measured on healthy, fully expanded leaves using a portable photosynthesis system (Walz GFS-3000, Effeltrich, Germany). WUE was calculated as Pn/Tr. For each plant, measurements were taken on healthy, fully expanded leaves at a comparable position.

#### 4.4.2. Pigment Content Determination

The relative chlorophyll content (SPAD value) was determined using an 80% acetone extraction method [36]. Briefly, the main veins were removed from the leaf samples. Subsequently, 0.2 g of fresh leaf tissue was weighed, ground in liquid nitrogen, and homogenized with 10 mL of 80% acetone. The homogenate was kept at 4 °C in darkness for 24 h to allow for pigment extraction. Following extraction, the mixture was centrifuged at 4000 rpm for 10 min. The supernatant was then collected, and its absorbance (A) at wavelengths of 665 nm, 649 nm, and 470 nm was measured using a UV-Vis spectrophotometer (Agilent Technologies, Santa Clara, CA, USA). The relative concentrations of chlorophyll a (Chl a), chlorophyll b (Chl b), and total chlorophyll were calculated according to the following equations:

(1) $$Chl\;a=12.21\times A_{665}-2.81\times A_{649}$$

(2) $$Chl\;b=20.13\times A_{649}-5.03\times A_{665}$$

(3) Chl=Chl a+Chl b

where A represents the absorbance of the sample extract at the specified wavelength, and Chl a and Chl b correspond to the relative concentrations of chlorophyll a and b, respectively.

#### 4.4.3. Mineral Element Determination

A 1.00 g sample of dried and powdered stem and leaf tissue was digested using the concentrated sulfuric acid-hydrogen peroxide (H_2_SO_4_-H_2_O_2_) digestion method [37] to completely decompose organic matter. After cooling and dilution of the digestate, an aliquot was taken for total nitrogen (N) determination using a Kjeldahl nitrogen analyzer (FOSS, Hillerød, Denmark). Another aliquot was used for total phosphorus (P) determination by the vanadomolybdate yellow colorimetric method at 440 nm wavelength. Crude protein (CP) content was calculated based on the nitrogen result using the formula: CP = N content × 6.25. The N:P ratio was also calculated to assess nutritional balance.

### 4.5. Data Processing

Prior to analysis, the normality of data distribution was tested using the Shapiro–Wilk test, and homogeneity of variances was tested using Levene’s test. If the data met the assumptions for parametric tests, one-way analysis of variance (ANOVA) followed by LSD post hoc test for multiple comparisons was performed. Correlation analysis was used to explore relationships between variables. All analyses were conducted using SPSS 26.0. Significant differences among groups are indicated by different lowercase letters in tables and figures. Charts were plotted using Origin 2024.

## 5. Conclusions

This study systematically investigated the growth and physiological-ecological responses of *A. fruticosa* to different shading gradients within a photovoltaic power station in the Kubuqi Desert, elucidating its adaptation patterns and coordinated mechanisms to the dynamic shaded habitat. The main conclusions are as follows: (1) Light Shading (LS) created the most favorable “light-water coordinated” microenvironment for the growth of *A. fruticosa*. Plant height, basal diameter, and crown width under LS were significantly greater than under both full sunlight (CK) and Heavy Shading (HS). In the early growing season, leaves exhibited morphological plasticity by expanding their area to adapt to the low-light conditions. (2) *A. fruticosa* adapted to the shaded habitat by optimizing its photosynthetic pigment composition and water-use strategy. Shading promoted the accumulation of total chlorophyll (Chl) and significantly reduced the Chl a/b ratio, driven by a substantial increase in Chl b content, thereby enhancing the capture of diffuse light. The LS treatment maintained a relatively high net photosynthetic rate (Pn) while effectively reducing the transpiration rate (Tr), achieving an efficient balance between carbon assimilation and water consumption, which led to a significant improvement in water use efficiency (WUE). (3) Moderate shading favored the accumulation and balance of nitrogen and phosphorus nutrients. The LS gradient resulted in the highest contents of nitrogen (N), phosphorus (P), and crude protein (CP) in *A. fruticosa*. The N:P ratio was maintained within an optimal range (4.06–5.03), providing a balanced nutritional foundation for plant growth and metabolism. (4) The adaptation of *A. fruticosa* to dynamic PV shading stems from the coordinated regulation of its morphology, photosynthetic apparatus, pigment synthesis, and nutrient metabolism. Its growth performance is closely linked to photosynthetic efficiency, water use, chlorophyll synthesis, and nutritional status, collectively forming an integrated adaptive response network. In practical terms, this study identifies a quantifiable optimal shading duration of approximately 6 h per day for *A. fruticosa*. This critical parameter provides a direct physiological basis for spatially configuring PV infrastructure and vegetation: planting should be prioritized in light-shaded inter-row zones, and panel spacing can be calibrated to approach this optimal shading target. Our findings thus offer actionable insights for enhancing the sustainability and synergy of “PV + sand control” projects in arid ecosystems.

## Figures and Tables

**Table 1:** Growth changes of *A. fruticosa* under different shading gradients.

Index	Time	HS	LS	CK
Plant height (cm)	July	42.67 ± 4.04 ab	47.33 ± 6.81 a	33.67 ± 5.86 b
	August	44 ± 3.61 ab	56.67 ± 10.5 a	38.33 ± 1.53 b
	September	46.33 ± 3.21 b	59.33 ± 10.02 a	41.33 ± 3.21 b
Basal diameter (cm)	July	0.81 ± 0.04 b	1.26 ± 0.25 a	0.54 ± 0.03 b
	August	0.86 ± 0.11 b	1.41 ± 0.26 a	0.78 ± 0.03 b
	September	0.98 ± 0.19 ab	1.25 ± 0.08 a	0.76 ± 0.13 b
Crown width (cm)	July	40.83 ± 11.84 a	47 ± 3.91 a	34 ± 6.38 a
	August	46.17 ± 11.25 ab	62.33 ± 8.62 a	43 ± 4.82 b
	September	49.83 ± 13.08 a	66.17 ± 12.41 a	54 ± 6.5 a
Leaf length (cm)	July	2.58 ± 0.04 a	2.40 ± 0.09 a	1.78 ± 0.25 b
	August	2.55 ± 0.39 a	2.39 ± 0.29 a	2.08 ± 0.24 a
	September	2.05 ± 0.31 ab	2.36 ± 0.18 a	1.79 ± 0.25 b
Leaf width (cm)	July	1.34 ± 0.08 a	1.09 ± 0.10 b	0.77 ± 0.12 c
	August	0.90 ± 0.28 a	0.85 ± 0.12 a	0.66 ± 0.06 b
	September	0.82 ± 0.22 a	0.9 ± 0.12 a	0.76 ± 0.12 a
Leaf thickness (mm)	July	0.16 ± 0.02 ab	0.15 ± 0.03 b	0.20 ± 0.01 a
	August	0.14 ± 0.01 b	0.16 ± 0.01 b	0.19 ± 0.02 a
	September	0.11 ± 0.01 b	0.13 ± 0.01 b	0.17 ± 0.02 a

Data are presented as mean ± standard deviation. Different lowercase letters within the same row indicate significant differences among treatments (*p* < 0.05).

## Data Availability

The raw data supporting the conclusions of this article will be made available by the authors, without undue reservation. The data are not publicly available due to copyright.
